# How the evolution of air breathing shaped hippocampal function

**DOI:** 10.1098/rstb.2020.0532

**Published:** 2022-02-14

**Authors:** Lucia F. Jacobs

**Affiliations:** Department of Psychology, University of California, 2121 Berkeley Way, Berkeley, CA 94720-1650, USA

**Keywords:** lungfish, olfaction, spatial orientation, theta, tetrapod, vomeronasal

## Abstract

To make maps from airborne odours requires dynamic respiratory patterns. I propose that this constraint explains the modulation of memory by nasal respiration in mammals, including murine rodents (e.g. laboratory mouse, laboratory rat) and humans. My prior theories of limbic system evolution offer a framework to understand why this occurs. The answer begins with the evolution of nasal respiration in Devonian lobe-finned fishes. This evolutionary innovation led to adaptive radiations in chemosensory systems, including the emergence of the vomeronasal system and a specialization of the main olfactory system for spatial orientation. As mammals continued to radiate into environments hostile to spatial olfaction (air, water), there was a loss of hippocampal structure and function in lineages that evolved sensory modalities adapted to these new environments. Hence the independent evolution of echolocation in bats and toothed whales was accompanied by a loss of hippocampal structure (whales) and an absence of hippocampal theta oscillations during navigation (bats). In conclusion, models of hippocampal function that are divorced from considerations of ecology and evolution fall short of explaining hippocampal diversity across mammals and even hippocampal function in humans.

This article is part of the theme issue ‘Systems neuroscience through the lens of evolutionary theory’.

## 1. Introduction

Models of hippocampal function are derived from intensive study of two rodent species, the laboratory mouse and laboratory rat. These two species are remarkably similar, taxonomically and ecologically. Both are from the subfamily Murinae (Family Muridae) in the Order Rodentia, a highly successful and diverse taxonomic group [1,2]. Both are small-bodied, nocturnal, quadrupedal omnivores. Yet focusing research on two species chosen for convenience, not science, demands that we question assumptions that these murine species can be stand-ins for all mammals or even all rodents or even all murines [3,4]. As the history of radical behaviourism has taught us, it is a mistake to assume that a learning process in one species can be extrapolated to species inhabiting different ecological niches [5], and this is true even of these two species [6,7].

The second problem is more problematic. If murines are to be the default model of spatial orientation by the hippocampus, then it logically follows that experimental design should be tailored both to the species and the brain structure. Until the discovery of hippocampal place cells revealed its role in spatial orientation [8], the hippocampus was described as the ‘nose brain’, the rhinencephalon [9]. Yet olfaction, a sensory modality that is both a phylogenetically conserved primary input to the hippocampus [10,11], and a primary sensory modality for murines [12,13], has rarely been studied in the context of spatial orientation in these species (but see [14–17]). This is slowly changing [18,19].

There are several reasons for this neglect. As diurnal apes, we are ‘blinded by vision’, studying the sensory modalities for which we have greater conscious awareness [9]. Events in human history have also contributed to human olfaction being underestimated and understudied [20,21]. Naturalistic spatial orientation to odours has been addressed in even fewer studies [22–24].

However, a second and more compelling reason is that olfaction remains mysterious, despite its universal importance in animals and its deep evolutionary history [25,26]. Many of the most fundamental questions in olfactory neuroscience remain unanswered [27,28], including the olfactory code. We lack essential models to predict the ‘smell’ of a chemical structure: the relationship between structure, olfactory receptors (ORs) and perception [29]. Thus, except for a few classic studies listed above, most experiments on murine spatial orientation use visual cues. As these experiments also demonstrate, visual cues are important even in the presence of olfactory cues. In fact, the two modalities may have clearly matched complementary advantages and disadvantages [30], but at the very least, our experiments should be designed in the explicit knowledge of the natural statistics of the sensory cues in the study species's environment.

Fortunately, we are in the midst of a conceptual crisis that threatens our complacency with the status quo, offering hope that future species will be chosen based on scientific principles [3,4]. The crisis comes from unexpected results that question the hegemony of visual cues in the study of hippocampal function. This is a series of prominent studies demonstrating that nasal respiration profoundly influences memory processes, in humans as well as murines [31–34]. Ironically, the phenomenon was first described in 1942, when the hippocampus was still known as the rhinencephalon. Studies of the European hedgehog had already identified the synchronization of theta and respiration, but until, recently, these oscillations were viewed as artefacts to be filtered from recording data [32,35].

This is not a question limited to olfactory structures processing odours, but instead the realization that nasal (but not oral) respiration entrains fundamental processes of cognition. Through slow network rhythms, in particular theta oscillations, nasal respiration appears to coordinate function across widely dispersed brain regions. This occurs both within and outside the limbic system, with wide-ranging effects in prefrontal and parietal cortices. This is not a case of the tail (respiration) wagging the dog (cognitive function). Instead, a recent study has demonstrated that the direction of causality is from theta to respiration and not the reverse. This study also found evidence for a peripheral reafference signal in gamma oscillations, however, [36]. This peripheral reafference as well as studies showing that oral respiration can play a role [34] must be reconciled in this emerging field.

Given that nasal respiration appears to be actively involved in human memory, the question is why? It is not a question of oxygen; oral respiration, which supplies a greater volume of air, has a significantly smaller effect [37,38], and here our focus on murines might be a good thing. If the olfactory system and the hippocampus are in fact a limbic navigational system [39], then using two highly olfactory model species is a lucky break. However to proceed further, to ask the right questions of these and other species, to identify the mechanisms, we need to understand the behaviour at multiple levels of analysis, including adaptive function and phylogeny [40,41].

Geneticist Theodosius Dobzhansky, a friend of my grandfather, Gregory Altschuller, wrote in 1973: ‘Nothing in biology makes sense except in light of evolution’ [42, p. 125]. I would add, echoing others [43], that nothing in neuroscience makes sense except in light of behaviour. Here, I will propose that the evolution of air breathing constrained the functions of the limbic navigational system, which I conceptualize as the integrated function of the main olfactory system (MOS) and the hippocampus in spatial orientation. I will argue that only by reconceptualizing hippocampal function can we not only understand why nasal respiration influences memory but also how olfactory and hippocampal functions have radiated and adapted to new challenges, even in our species.

## 2. Return to the rhinencephalon

I begin by summarizing my previous three syntheses on these questions, integrating concepts from ecology, evolution, cognition and neuroscience. This is my perspective on the function and evolution of the hippocampal limbic system. Reader beware—limited space precludes contrasting my own hypotheses with other models of hippocampal function. I likewise openly acknowledge that my own models have yet to become widely influential. But given the biases associated with standard models of hippocampal function, the reader is forewarned that I do not apologize for my countervailing bias that olfactory evolution is fundamental to understanding how the brain works.

### (a) The parallel map theory

In this model, Francoise Schenk and I proposed to ‘unpack’ the cognitive map by distinguishing between stimuli that are perceived as gradients or compasses (such as odour plumes) and stimuli perceived as objects [44]. Once we did this, it became clear that the two components of the mammalian hippocampus, the dentate gyrus and the Ammon's horn, subserve different functions. We proposed that the ancestral hippocampal homologue of vertebrates, with its conserved relationship to the olfactory system and the septal nuclei, relied primarily on compass, also known as directional cues, such as odour gradients. That in the more derived hippocampus seen in mammals, an independent system had evolved that encoded space in arrays of discrete objects, also known as positional cues. Hence the ancestral hippocampus oriented to internal directional cues (path integration) and external cues such as gradients of odours, sound or light, as well as physical features that polarized a space, such as the geometry or slope of a space [45–48].

When such gradients intersected, we proposed that this could create a coordinate system, which we called the bearing map. This could define a location in the absence of positional cues. We proposed that the bearing map was mediated by the dentate gyrus and represented the sum total of an individual's lifetime explorations, its expansions encoded via adult hippocampal neurogenesis. Finally, because encoding gradients should require the ability to measure the distance between iterated samples, we proposed that hippocampal theta oscillations could act as a pacemaker to measure the distance along such a gradient. We defined positional cues as unique, discrete objects, that are coded individually and encoded in arrays as sketch maps, mediated by the CA1 subfield of Ammon's horn. By contrast to the bearing map, we proposed this map was a non-Euclidean, topological and ephemeral record of a specific positional cue array. Sketch maps were proposed to be encoded in discrete learning events as the navigator experiences new events in space and time.

In addition, while the maps can operate independently (e.g. after subfield inactivation), the intact hippocampus could also combine these parallel maps. Here the ancestral bearing map would calculate a low-resolution map based on directional cues and the more derived sketch maps would add high resolution details from positional cues. Such a complete spatial representation of the environment, formed by recoding new sketch maps into the permanent bearing map, would allow for flexible re-routing, i.e. cognitive mapping, which would be mediated by the CA3 subfield [49].

The parallel map theory gave a theoretical framework to understand cognitive sex differences in humans and mice [45,50], even specific patterns in hippocampal synaptic plasticity [51–53]. The parallel map theory remains unique for its basic tenet that hippocampal function is scaffolded on distributed stimuli, such as odour gradients.

### (b) The olfactory spatial hypothesis

The parallel map theory suggested a reassessment of the primary function of vertebrate olfaction, from identity *per se* to identity in the service of spatial orientation [39]. I proposed that one reason the olfactory code has not been cracked is because of the assumption that odour identification is the primary and overarching function. An odour can simply identify or that identity can used to establish the meaning of a positional cue (e.g. scent mark) or a directional cue (e.g. plume). Odours in biological systems are complex [54–56]. Thus an odour's use may begin with diagnosing the identity, then, by encoding its spatio-temporal context (as a positional or directional cue), derive its meaning to the receiver.

Olfactory psychophysics might also function to encode space. For example, one complex phenomenon is the perception of an odour mixture. In some cases, the individual odourants can be discriminated (elemental perception); in others, only the mixture is perceived (configural perception) [57,58]. In ethological interpretation of these phenomena, I proposed that elements and configurations might act independently or together, in a similar parallel map architecture. If so, then this could explain similarities in function between the hippocampus and the piriform cortex [59]. Likewise, following the gradient of concentration (directional utility) where the percept changes abrubtly, could create spatial structure in the form, creating what I called neighbourhoods of odour similarity, concordant with concentration and distance.

Studies of the neural basis of spatial olfaction in murines and humans are emerging to support the thesis of the olfactory spatial hypothesis [19,56,60,61]. For example, in a recent summary of the field, Marin *et al.* [19] proposed that the concept of odour spatial neighbourhoods could explain recent results of a grid-like pattern in human piriform cortex [62]. Also in humans, hippocampal activation and virtual maze performance correlates with olfactory discrimination accuracy [63]. In laboratory mice, hippocampal place fields can be driven by odour alone [18]. Also in the laboratory mouse, a recent study has shown that the posterior piriform cortex creates ‘odour-place’ maps [64].

### (c) Spatial olfaction in humans: the navigational nose hypothesis

A corollary of the olfactory spatial hypothesis is that if a species is orienting to odours, this should predict related morphological adaptations [24]. Across the animal kingdom, invertebrates have evolved paired structures, such as antennae and tentacles, to enhance their accuracy of spatial orientation using stereo olfaction [28,65,66]. Terrestrial vertebrates have likewise evolved structures such as external noses to enhance odour sampling and stereo olfaction [67,68].

In the navigational nose hypothesis, I proposed that selection for spatial navigation shaped the evolution of olfaction in our own genus, *Homo*, leading to the evolution of the only external pyramid (external nose) in apes and other hominids*.* Our genus *Homo* was the first long-distance ape, expanding its range across continents and evolving a suite of adaptations for efficient long-distance locomotion [69]. I proposed that this led to selection for increased navigational accuracy, which could have been achieved by adding stereo olfaction to the hominin navigational toolkit.

As in vision and audition, stereo olfaction increases the accuracy of orientation. This has been demonstrated in insects (honeybee, [70]) and mammals (laboratory rat [71], common mole [72]) and humans [22,73,74]. In sharks, an increased distance between incurrent sensors increases the delay between odour arrival in each nostril and enhances accuracy of orientation, and may underlie the evolution of head shape in bonnet-headed sharks [75]. Computational models of stereo olfaction show that stereo increases the efficiency of odour plume tracking [60,76].

Modern human nose shapes are diverse. The navigational nose hypothesis explains this as a correlate of space use. The development of Neolithic agricultural economies increased sendentism, population density and infectious disease [77,78]. Narrower (leptorrhine) nasal morphology is associated with enhanced olfactory discrimination [79]. Hence this change in human economies would have selected for narrower noses to optimize diagnostic, not directional, olfaction under these conditions.

No other hypothesis to explain the evolution of the external pyramid in the genus *Homo* posits an olfactory function. The standard hypothesis, proposed a century ago, concerns the nose's other function, respiration [80]. Many of the assumptions of this hypothesis are now in dispute [81,82]. The navigational nose hypothesis thus presents an important alternative hypothesis, and one that places the question squarely within comparative biology and the general principles of sensory evolution.

If adaptations for spatial orientation to odours have driven the evolution of humans, and the human nose is now driving basic memory processes, then the next question is how did this evolve.

## 3. The evolution of air breathing

Olfaction, air breathing and indeed the first vertebrates evolved in the ocean; our mammalian brain and skeleton have hardly deviated in fundamentals from the fish Bauplan [10,83,84]. We exist in a clade with other fishes: we are the Osteichthyes, vertebrates with internal bony skeletons. This includes the ray-finned fishes (the *Actinopterygii*) and the lobe-finned fishes and tetrapods (the *Sarcopterygii*), while vertebrates with cartilaginous skeletons (sharks, rays and holocephalans), constitute the Chondrichthyes [85]. To understand how air breathing and olfaction has influenced the evolution of our Osteichthyan brain, first we must understand how olfaction in water differs from olfaction in air.

Fishes use chemosensory systems to detect resources, such as conspecifics, prey habitats and threats, such as predators [86,87]. The fish MOS can encode a large range of molecular sizes, from amines associated with food to entire peptides. By contrast, a terrestrial MOS is limited to small, volatile molecules that can be transported in air [88]. Another advantage of aquatic olfaction is spatial scale. Because of the refractive index of water, olfaction is a superior distance sensory modality than aquatic vision [89]. Thus olfaction is critical for spatial orientation underwater. For example, catfish orient to prey by integrating inputs from the MOS with the lateral line system to track odours in the prey's wake [90]. Even seals, a secondarily aquatic mammal, can track prey by the hydrodynamic patterns of wake with their vibrissae [91].

But most important, spatial olfaction in fishes is independent of respiration. Oxygen exchange occurs in the gills and other organs [92], but olfaction is mediated by an independent, dedicated olfactory organ. Bony fishes have two pairs of nostrils, the anterior pair for incurrent flow to the nasal capsule and olfactory epithelium, and the posterior pair for excurrent flow after the odourants have been sampled [86]. Thus in most fishes, unlike terrestrial tetrapods, oxygen exchange is structurally and functionally independent from olfaction.

In summary, olfaction in fishes is a high-capacity sensory modality, able to detect a wide range of molecular sizes, the ability to detect odour sources from a great distance and all of this done with a stand-alone system. Small wonder that chemosensory systems for foraging, predation, social interactions and reproduction are widespread underwater [88].

### (a) Nasal respiration and spatial orientation

The independence of olfaction and respiration is present even in fishes that breathe air. Air breathing is widespread in fishes, having evolved independently at least 32 times, with over 400 air-breathing species currently living [92]. Fish taxa vary greatly in how the aerial oxygen is acquired, from the air gulping and two-stroke buccal pumping seen in lobe-finned lungfish [93] to inspiration through dorsal spiracles in the bichir, a basal ray-finned fish [94] to cutaneous oxygen exchange and exchange via the secondarily evolved swim bladder [92].

But evolution of the lung was an early and important innovation, occurring at least 400 Ma, at the Silurian–Devonian border. The appearance of the lung coincided with a major re-engineering of the cardio-pulmonary system, allowing freshly oxygenated blood to be shunted to the heart before peripheral tissues, for the first time in vertebrate evolution. This would have increased aerobic capacity and fuelled an increase in behavioural complexity and activity [95,96]. Lungs have been secondarily lost in many fish taxa since then, evolving into new functions such as swim bladders, for example, in the highly successful teleost lineage [97].

New evidence on the evolution of lungs has come from the first complete genome sequencing of two lungfish genera (whose genome size had previously forestalled this effort) and representatives of living basal ray-finned fish lineages, including the bichir. These studies confirm earlier work that lungfish are the fish lineage most closely related to tetrapods [98,99]; but these comprehensive genomic studies of these basal ray-finned fish lineages also found homologies with tetrapod genes involved in limb development, cardiac function and lung function [85,99–102].

However, despite widespread air breathing and genes that later will be coopted into adaptations for the tetrapodomorph fish transition from water to land, only two groups use nasal respiration. We are one, or rather we are the direct descendents of the group that became the tetrapods, the tetrapodomorphs and the other is our close relative, the lungfish, the lineage of dipnomorphs. Both of us are lobe-finned fishes, Sarcopterygians [85].

Based on the palaeontological record, only these two groups evolved the necessary structure for nasal respiration. These are the choanae, or internal nostrils, which allow inspired air to pass from the external nostril through the internal nostril to the lung. The paired choanae in these groups are the homologues of the paired posterior nostrils of bony fishes described earlier. By studying changes in the posterior nostril over evolutionary time, the lateral movement of the posterior nostril could be documented, as it migrated laterally then ventrally, reaching the edge of the mouth and finally becoming located within the palate. The key discovery of an intermediate stage of nostril migration in *Kenichthys*, a fossil lobe-finned fish, established this homology in tetrapodomorphs [103]. At the same time that choanae evolved in tetrapodomorphs, the posterior nostril in the lungfish lineage was also migrating, also becoming choanae [104]. Thus nasal respiration—in essence the first nose, where inspired air passes over olfactory epithelium before being sent to the lungs for oxygen exchange—only evolved twice, in our lineage and that of lungfish.

This history raises a question of the selective advantage of the initial lateral movement of the posterior nostrils, as the respiratory advantage could only be realized as choanae. Any intermediate step must have conferred an evolutionary advantage, especially as it appeared independently in two lineages. Per Ahlberg has suggested that the lateral movement could have increased water flow through the nasal capsule, increasing olfactory capacity (P. E. Ahlberg 2021, personal communication). A possible mechanism for this increased flow might be that proposed by Jonathan Cox, in his review of the hydrodynamics of fish olfaction. He describes several mechanisms by which water flow over the nares would increase water movement within the nasal capsule. One of these is a mechanism similar to a Pitot tube, where a more lateral position of the posterior nostril would have this effect. Such a Pitot-like mechanism could theoretically increase flow through the nasal capsule [86]. If so, this might explain the initial movement of the nostrils in fish lineages, leading eventually to the evolution of the internal nostril in lungfish and the tetrapod lineages, and hence the independent evolution of nasal respiration.

Thus our two lineages are unique as there is no such evidence for nasal respiration in other living species of air-breathing fishes. For example, the bichir respires through dorsal spiracles, a trait found in earlier fish groups [94]. This is important because the recent genomic analysis of the bichir and other air-breathing basal ray-finned fishes reported the presence of ORs in the OR gene family that is associated with terrestrial, not aquatic, vertebrate species [99,100,102]. The studies conclude that this provides evidence for olfaction of airborne odours in these species. This conclusion may be premature, as these gene families are also found in non-air breathing fishes, such as the deep sea coelacanth [85].

I agree with Ahlberg. As reviewed by Touhara *et al*. [29], we lack data and hypotheses to identify the ligands of most ORs, and thus identifying a gene family cannot specify which odourants are being perceived. Even in mammals, we lack hypotheses to explain the distributions and number of ORs from different gene families across species. Finally, not all ORs are olfactory but some are located outside the olfactory system, subserving as yet unknown functions [29]. So the presence of genes in ‘terrestrial’ OR gene families in a fish species that lacks narial respiration cannot be evidence for olfaction of airborne molecules.

But I would like to suggest another possible interpretation of these genomic results. Perhaps the ORs in these ‘terrestrial’ gene families are encoding not airborne odours but aqueous odours associated with resources on or near land, such as terrestrial materials leaching odours into the water of the near shore habitat in which tetrapods evolved. Later, there would have been strong selective pressure for these OR gene families to be expanded even further in early tetrapods, once they could encode odours in air and water.

Such hybrid noses are not unknown but are found in secondarily aquatic vertebrates, which orient to airborne odours to locate land resources. Loggerhead sea turtles orient to air-borne odours emanating from islands [105] and green sea turtles are preferentially attracted to the airborne odours of island mud [106]. Diverse vertebrate species (sea turtle, seabirds, harbour seal, bowhead whale) orient to the airborne odour of dimethyl sulfide, a metabolic byproduct of phytoplankton that is associated with fish schools that are otherwise difficult to locate [107–111].

This interpretation of the genomic data would complement hypotheses from palaeontology about the ecological habitat of the water–land transition, for example the intertidal hypothesis [112]. Derived from the palaeontology of early trackways (footprints), this hypothesis proposes that tetrapods evolved in shallow marine intertidal and/or lagoon habitats, where the twice daily tides would have provided a predictable source of plant- and animal-derived nutrients. Thus the intertidal zone would have served as a transition step, providing resources to support an experimental proving ground for early tetrapod innovations [112].

The olfactory landscape of such an intertidal or lagoon environment would have included the odours of nutrients dissolved in water but also nutrients carried to land and exposed to air at low tide. Perhaps this could explain some of the patterns of ORs, with genes from diverse gene families, in these basal ray-finned fishes. I offer this as speculation, of course. In any case, only two fish lineages, our tetrapodomorph lineage and our cousins the lungfish, evolved internal nostrils for nasal respiration and did so independently—but only one of us ended up on land, perhaps led there by our noses.

## 4. The appearance of a second olfactory system

Now the stage is set. Tetrapodomorph, air-breathing lobe-finned fishes are leaving their footprints in the mud as they come onto land [112]. They are breathing air and sniffing odours through the nares, using choanae. What these lineages with choanae also have is a second olfactory system, the vomeronasal system (VNS), also called the accessory olfactory system [113].

The evolution and function of this system are still not fully understood [114]. In murine rodents, it is clear that a primary function of the VNS is to encode pheromones [115–118], but the VNS is found across vertebrates [113,114,119,120]. Even within mammals, the VNS does not encode all pheromones [121] and even within rodents, VNS function can vary between closely related species [114]. In short, lacking a unifying hypothesis to explain variation in VNS function within mammals or across vertebrates, it is unclear why this second olfactory system evolved.

Nasal respiration may hold the key. The receptors for the VNS are found deep in the fish lineage, found in cartilaginous fishes [122] and as far back as jawless fishes such as the sea lamprey [123]. But the first VNS circuitry that is homologous to that found in tetrapods is seen in the lungfish [124]; i.e. the only other lineage with nasal respiration. Anatomically, the lungfish nasal capsule exhibits two kinds of sensory epithelia, one similar to the MOS and the other similar to the VNS, similar to the semi-aquatic African clawed frog (*Xenopus*) [125]. It is also analogous to that of sea turtles, which have a highly complex internal nose for aqueous and airborne odour perception [126,127]. Both *Xenopus* and aquatic turtles (green sea turtle, soft-shell turtle) have surprisingly large OR gene numbers, which has been ascribed to this dual function nose [128–130].

Why did a second olfactory system evolve in this way? Odours encoded by the VNS are more likely to be large, non-volatile molecules found on a solid substrate (e.g. scent mark) [113]. These molecules are then transported, often in an aqueous fluid such as saliva, to the vomeronasal organ, which opens into the mouth. While the MOS is tuned to a broad range of odours, both biotic and abiotic, the VNS, tuned to the odours of biotic agents [131], is much more variable [132–134]. Hence the VNS is used by vertebrates to diagnose a biotic agent's physiological state, whether this is its reproductive, emotional or disease state [135].

This may explain why it evolved when it did, in the nasal respiration lineages. The terrestrial MOS is constrained to small molecules carried on air currents from remote sources, as small volatile molecules can be carried over long distances in the air, even hundreds of kilometres [56,109,136,137]. Evolving from a fish MOS where molecular size is not a constraint, a tetrapod MOS specialized in airborne odours would be ideally suited for detecting remote resources—i.e. maximizing its spatial orientation function. This would explain why in tetrapods, MOS allometry and function tally so closely with a species's need for flexible re-routing in space [39].

By contrast, odours encoded by the VNS are sampled by physical contact, where spatial location is unambiguous. Here the function of the VNS would be to replace the MOS function seen in fishes, of encoding large molecules in aqueous mixtures. Coming to land and re-engineering the MOS for airborne odours would have left a serious vacuum in a species's olfactory capacity. Large molecules and complex odour mixtures represent a critical source of information to terrestrial animals [54,56,138].

This hypothesis has important implications for olfactory evolution in vertebrates, which I am exploring in a separate work (the PROUST hypothesis: perceiving and reconstructing odour utility in space and time [139]). The PROUST hypothesis predicts a disassociation between functions—the MOS specialized for directional olfaction (determining the spatial distribution of an odour) and a VNS for diagnostic olfaction (determining the functional category of an odour). This disassociation has been demonstrated experimentally in two distantly related tetrapod groups, the house mouse (the ancestral species of the laboratory mouse) and in a species of garter snake, described below.

The scent mark of the house mouse is comprised of volatile and non-volatile components that provide different information [140]. A female mouse requires physical contact initially with the large molecules, e.g. the major urinary protein, to deduce the male's identity and current physiological state [141]. Airborne odourants are encoded by the MOS and associated with the diagnostics by the VNS encoding of identity and state. Once this association has been made, a female can identify a male safely and remotely, by airborne volatiles [140–142]. This has important survival value: strange males represent a significant threat of infanticide to breeding females [143], so the MOS allows a female to detect and avoid such threats at a safe distance.

The PROUST hypothesis could explain patterns of brain activation in the laboratory mouse. Functional imaging revealed that odours of subordinate males activate the MOS of the female mouse while the VNS is activated by odours of dominant male mice [144]. An ethological interpretation of this result could be as follows: because a subordinate represents an immediate threat of infanticide, the important use is not his identity but his location, which, as a spatial function, activates the MOS. By contrast, the use to the female of the dominant male's odour, whose location is always known, is not spatial (MOS) but state (VNS)—his current level of stress, disease, nutrition, i.e. the parameters a female must weigh to determine the value of a male [135]. Hence the odour of the dominant male primarily activates the female VNS [139].

A similar pattern is seen in garter snakes: a disassociation of directional olfaction by the MOS and diagnostic olfaction by the VNS, in the context of the predation of earthworms. Each arm of a Y maze was marked with a different form of earthworm extract, either a liquid (or dried) trail on the maze floor or earthworm extract that had been atomized and was airborne. As in house mice, the MOS was necessary and sufficient to track airborne odours of this prey while the VNS was necessary and sufficient to track the substrate-bound trail. Only the VNS function required physical contact with the extract [145,146].

In summary the PROUST hypothesis proposes that with the invasion of land, tetrapods split the work of the ancestral fish MOS into spatial orientation by the MOS and biotic agent recognition by the VNS. What then followed was the adaptive radiations of these two systems in vertebrates [139]. Now that I have laid out my ideas on the evolution of nasal respiration and the function of the MOS, we can proceed to the hippocampus.

## 5. Adaptive radiation in hippocampal functions

### (a) The hippocampus of ancestral mammals

Even before true mammals emerged, mammaliaform groups were radiating widely [147]. Their early innovations profoundly changed their sensory ecology. The ecological niche that mammals captured early on was nocturnality [148], an activity pattern niche that strongly favours olfaction [13]. Next, with the evolution of secondary miniaturization of the body, early mammals evolved innovations in jaw morphology and bite force, which freed up jaw bones and allowed the evolution of the mammalian inner ear [149]. The successful early mammal formula was thus a tiny nocturnal species, with innovative olfactory and auditory systems. During this period of innovation in the Jurassic early mammals, there were also notable grade shifts in brain size. Each shift was preceded by an increase in the relative size of the MOS [150]. A similar pattern has been documented in early Miocene primates, where endocasts show an increase in MOS volume that preceded grade shifts in whole brain size [151]. In living mammals, such grade shifts are found in species with increased space use [39]. Thus, it is possible that increases in brain size were initiated by increased space use in an olfactory landscape.

Small terrestrial mammals orienting to odours in space using the MOS should also need to measure the carrier fluid of the odour, i.e. the velocity and direction of the air current. This has been demonstrated now in laboratory rats: they can measure wind direction from vibrissal inputs [152]. Like fishes following the wake of prey, homing pigeons learn the association between odour and wind direction in their home loft and use this retained information later to navigate home from novel locations [136,153–155].

Thus the laboratory murine may in fact possess all the mechanisms of early mammals—olfaction, audition, vibrissae—needed to map an odour gradient. This integrated system has been the subject of elegant neuroscientific research, demonstrating a synchronization of nasal respiration, whisking (vibrissal movement) and hippocampal theta oscillations [156]. Thus murines synchronize the chemotactic inputs (the sniff, delivering the sample to the MOS via nasal respiration) and anemotactic inputs (the wind direction, as perceived by the mystacial vibrissae), exactly as catfish integrate the odour and hydrodynamic trails of their prey [90]. In the parallel map theory, the only remaining input to encode a hippocampal bearing map would be the theta oscillations to act as a pacemaker [44]. This, too, is synchronized with sniffing and whisking in laboratory murines, and this might have been similar to the sensory world of mammals for many million years. But mammals did not stay small and terrestrial, and we should be able to find the exceptions that prove the above rule in those species who radiated away from this ancestral sensory niche.

### (b) Adaptive radiations in hippocampal theta

I begin with a caveat from an expert, which I am not: ‘The theta rhythm is a neuroscience enigma’ [157, p. 1]. The study of hippocampal theta oscillations is characterized by a rich, interdisciplinary, controversial literature of empirical and theoretical studies [158]. With prominent models in conflict [157,159], I propose here a new approach, an ethological analysis, which has been neglected.

In the parallel map theory, for a terrestrial mammal to build its bearing map from odour gradients, it must collect iterated sniff samples along an odour gradient, marking self-movement distance with hippocampal theta and synchronizing the olfactory input with independent measures of wind direction from vibrissal input to represent the plume [44]. Thus what the parallel map theory would contribute to this debate is to specify that the key parameter determining species differences in theta is their reliance on directional olfaction. If hippocampal theta underlies the ability of the hippocampus to measure self movement along an olfactory gradient, then patterns of theta should be predictable by a species's use of such gradients.

In 1972, Winson ended his review of species differences in theta calling for future research on ecological correlates [160]. In the first study of hippocampal place fields in bats, Ulanovksy and Moss likewise proposed that species differences in theta oscillations could be explained by ethology [161].

Patterns of theta should be analogous to neuroecological studies of the hippocampus in birds and mammals, where structure can be predicted from the spatial demands of an ecological niche [41,162,163]. Additional ecological factors should include trophic level (i.e. predator or prey), physical habitat and activity pattern. Hippocampal spatial mapping functions in diurnal species would, obviously, rely more heavily on visual stimuli. Nocturnal species should rely more heavily on olfaction and audition, as higher humidity and lower ambient temperature enhances the accuracy of following an olfactory trail [164]. Finally, there may be specific signatures found in crepuscular species (active at dawn and dusk), who maximize activity when neither visual nor olfactory cues are fully reliable [13].

We have only a paucity of species with which to test these predictions, moreover species not selected for comparative analyses [3,4]. Nonetheless, there are striking differences in the pattern of theta oscillations among mammalian species. These can be classified into four sensory ecological categories. First, there are five small terrestrial prey species: the nocturnal murine rodents (Order Rodentia; laboratory mouse, laboratory rat); the crepuscular Mongolian gerbil, from the same superfamily (*Muroidea*); the crepuscular guinea pig, from a different rodent suborder (*Caviomorpha*) and finally, from a different order, the crepuscular domestic rabbit (Order Lagomorpha). Second, there are two bat species, Order Chiroptera, one from each suborder (big brown bat, *Yangochiroptera* and the Egyptian fruit bat, *Yinpterochiroptera*). Third, there are two crepuscular predator species from the Order Carnivora: the domestic cat (Family Felidae) and the domestic dog (Family Canidae). Fourth, there are two species from the Order Primates, from two families in the diurnal catarrhine group, the human and the rhesus macaque.

One differences among species is in the relative frequency of two forms of theta that can be defined during behavioural testing. Type 1 theta is expressed continuously while an animal is engaged in voluntary locomotion; it is also atropine resistant. Type 2 theta is abolished by atropine treatment and is not dependent on voluntary locomotion, but is most often expressed while the animal is stationary [165]. While both types have been identified in most species, including laboratory murines, there are species differences in the prevalence of each type. There are also species differences in the behavioural context that is associated with each type. For example, Type 2 is less frequently observed in the laboratory rat but is easily activated in the domestic cat. It is particularly activated in the context of a stationary cat staring at a distant stimulus. This is a response that is not seen in the laboratory rat, even though immobility is a common strategy in prey species, to reduce predation risk, and ambush predator species, to reduce detection by prey.

Analysing species differences in the framework of the parallel map theory, Type 1 theta should be seen in species orienting to olfactory gradients. By contrast, a species that can measure distance from a stationary position should predominantly show Type 2 theta, as movement is necessary to use olfactory gradients for distance measurement. Thus these species must use another sensory modality to measure distance, such as stereo vision or audition.

The data summarized by Robinson [166] may reveal an association of theta type with sensory ecological niche. As predicted, nocturnal or crepuscular species that track odours across space while locomoting show a preponderance of Type 1 theta. These are: the laboratory mouse, laboratory rat, Mongolian gerbil, guinea pig and domestic dog. Second, species that orient to targets from a stationary position, relying instead on stereo vision or audition, show a preponderance of Type 2 theta: the domestic rabbit and the domestic cat. The rabbit, a prey species, uses stereo audition and specialized elongated pinnae to detect predators while immobile. The domestic cat is a felid, which, in contrast to a coursing predator such as a dog, is specialized as a solitary ambush predator [167]. The domestic cat shows a high amplitude Type 2 theta while alert, staring and immobile, similar to the domestic rabbit. Both species are collecting spatial information from a fixed location. By contrast, the domestic dog, another predator species, also employs vision but only at the end of the chase, instead relying almost entirely on directional olfaction while tracking prey [164]. Neither Type 1 nor Type 2 theta is seen in either bat species during active locomotion or flight, which I will address in the next section. Finally, the two diurnal primate species, highly visual species with hippocampal view fields that can be evoked from a stationary position [168], elicit theta oscillations that are dissimilar to Type 1 and more similar to Type 2, thus more similar to the domestic cat and rabbit.

Based on these few species, the patterns support the parallel map prediction that Type 1 theta functions in the context of directional olfaction during the active tracking of an odour gradient. I would interpret Type 2 theta instead as functioning in diagnostic olfaction, where nasal respiration is used to identify an object. This would be similar to the female house mouse using her MOS to identify the identity (species, sex) of an approaching threat. Without movement, however, the distance could not be measured. This may be why speed of locomotion and the physical scale of the environment appear to influence the accuracy of spatial mapping [48]. However, this would also offer an ethological functional explanation for the well-known phenomenon of changes in theta with locomotory speed [169]. A navigator moving more quickly along a gradient must adapt its measures of distance to speed. Orienting to an olfactory gradient requires the simultaneous samples of the odour and the odour vehicle, in this case air, which are exactly those features that have been demonstrated in laboratory rats [152,156]. Because both types of theta can be measured in murines, it should be possible to design experimental disassociation of directional and diagnostic olfaction tasks to test these predictions within a single species.

### (c) The hippocampus in extreme environments

The many innovations of Class Mammalia led to adaptive radiations into diverse and extreme environments, including the inhabiting of air and water [147,148]. Secondarily aquatic mammals include marine mammals such as the seals, whales and manatees [170]. Bats took to the air, occupying the unique niche of a mammal with powered flight, only the third vertebrate group after pterosaurs and birds to do so [171]. What then evolved multiple times in bats and once in toothed whales was a new mechanism to orient in space, echolocation [172]. Both groups evolved echolocation and this coincided with a loss of directional olfaction. I propose that it was this sensory substitution of directional olfaction by directional audition that led to the dramatic changes in hippocampal structure and function in echolocating toothed whales and bats.

Pioneering studies of hippocampal function in bats, both crawling and free-flying, using species from both suborders (the Yinpterochiropteran Egyptian fruit bat and the Yangochiropteran big brown bat) have identified clear hippocampal place cells and entorhinal grid cells [161,173]. Yet this cellular activity occurs in the absence of theta oscillations, which had been previously modelled as a necessary input for place and grid cell formation [161,173–175].

However, such a loss of hippocampal theta should be predicted from the sensory world of an echolocating bat [171,176]. Laryngeal echolocation (vocal emission) has evolved multiple times, in both suborders of bats [177]. Lingual echolocation (tongue clicking) has only evolved in one genus in one family, the genus *Rousettus* in the family Pteropodidae (fruit bats) [172]. The size of the olfactory limbic system (i.e. hippocampus and MOS) are relatively larger in the Pteropodidae than in the laryngeally echolocating bats [178], a group which now includes bats from both suborders [172]. Laryngeally echolocating bats also have low or absent adult hippocampal neurogenesis, compared to species in the Pteropodidae [39]. The lack of neurogenesis and the lack of theta during navigation—all of this would be consistent with the loss of the olfactory inputs to a hippocampal bearing map as posited by the parallel map theory.

Why bats do not use odours to navigate is something of a paradox, however, as both suborders of bats rely heavily on the MOS for diagnostic olfaction in social behaviours and foraging [176]. Although plumes in air are more turbulent than those in water [179], this has not constrained the use of odours in navigation by birds, the only other living vertebrate group capable of powered flight. Olfaction in birds plays a critical role in long-distance navigation in diverse species, from songbirds to seabirds [180–183] and the hippocampus and the olfactory bulbs increase in relative volume with space use in birds [39]. The parallel map theory may also reconcile differences between birds and mammals. The standard model of avian navigation is an integration of two maps: a non-hippocampal ‘navigational map’ and a hippocampal ‘familiar area’ map [184]. By re-examining the avian literature, it is possible to reconcile this difference between birds and mammals. In this scenario, the avian navigational map is an olfactory bearing map and the avian local area map is a multisensory sketch map. Hence, the hippocampus, a homologous brain structure in birds and mammals, would employ a similar computational architecture to solve novel shortcuts across space [48].

Back to bats: perhaps the reason why olfactory navigation, common both in terrestrial small mammals and birds, is rare or absent in bats is owing to a phylogenetic constraint. Because of their unique evolutionary history, respiration in bats is synchronized with wingbeat. Because of the unique respiratory system of birds, their wingbeat is independent of respiration [185]. Thus theoretically birds should be capable of dynamic sniffing, sampling odours strategically and independently of locomotion. Mammals such as domestic dogs and laboratory mice rapidly adapt their sniff frequency and intensity in a diagnostic and/or directional olfaction task. Increasing sniff intensity, for example, increases the contrast between the target and background [186–188]. Since birds travel at high speeds over long distances, this would be the ideal condition for an accurate olfactory bearing map [48] and could explain why avian long-distance navigators rely heavily on olfactory navigation. By contrast, the bat, with respiration synchronized with wingbeat, could use dynamic sniffing in a diagnostic task (e.g. discriminating conspecifics at a roost) but not for directional olfaction while navigating.

Like bats, whales (Order Cetacea) are another highly derived mammalian order, with sensory ecologies adapted for a completely aquatic life history. The order is comprised of the baleen whales (Suborder Mysticeti) and toothed whales (Suborder Odontoceti: dolphins, porpoises, killer whales) [170]. Like bats, whales have secondarily invaded a sensory niche hostile to olfaction, particularly to directional olfaction. No aquatic mammal has regained the ability to sample aqueous odours using the MOS [126]. This rules out the use of the MOS to track odours dissolved in water in these groups. Instead, one suborder, the toothed whales, have evolved echolocation, in a striking evolutionary convergence with bats, even to the point of convergent genetic mechanisms [177,189,190].

Toothed whales are also the only mammals with a vestigial MOS [126]. This is not only unique in mammals but almost unique among all vertebrates; the MOS is vestigial only in the secondarily aquatic sea snakes. But unlike the whales, sea snakes retain and employ the VNS to detect aqueous odours associated with prey [126]. Toothed whales have not only lost both olfactory systems (MOS, VNS) but they are also the only tetrapod group that is monorhinal. The paired nostrils found in all other vertebrate groups (except in *Agnatha*, the jawless fishes, such as the sea lamprey) is fused into a single blowhole, which functions only in respiration [170]. The surprisingly small dolphin hippocampus has been documented for over a century [191]. The cetacean hippocampus is between 8% and 20% of the size expected for a mammal of its brain size and, not surprisingly, lacks adult neurogenesis [192]. Yet an older study concluded that the entorhinal cortex was not reduced in size [193].

By contrast, baleen whales (suborder Mysticeti) have lost the VNS but have retained paired nostrils. They also retain MOS structure and olfactory functions. Bowhead whales employ the MOS to detect dimethyl sulfide, the krill metabolite mentioned earlier [111]. The retention of some MOS function might explain the difference in auditory systems between the two suborders. Although both suborders are highly vocal, sound production in the baleen whale is used solely for communication. Sound production in toothed whales is used both for communication and spatial orientation [194].

The parallel map theory would explain this as the exception that proves the rule: the only vertebrate group that has lost both olfactory systems and paired nostrils also shows a dramatic decrease in hippocampal size. This result cannot be explained by murine models and yet this re-engineering of the vertebrate brain Bauplan is associated with an extraordinary complexity of behaviour including spatial movements [194,195].

In summary, two unrelated mammalian taxa, having radiated into habitats hostile to olfaction (whales) or with phylogenetic constraints that disallow dynamic sniffing (bats) have converged dramatically, independently and multiple times (in the case of bats) on the solution of echolocation, one in air and one in water. Both show reduction or loss of the MOS, compared to terrestrial mammals of the same body size and loss and/or atypical dynamical behaviour in the hippocampal formation. These groups, both characterized by sophisticated three-dimensional spatial navigation abilities, present a serious challenge to the murine model of hippocampal function.

## 6. Conclusion^1^

To understand nasal respiration, we begin with oxygen. The lungs and aerial respiration that evolved in our Osteichthyan ancestors were accompanied by a redirection of blood flow, from the lungs directly to the heart [96,196]. This direct oxygenation of heart tissue could have supported further behavioural complexity, including increased space use and spatial cognition (§3). With the evolution of the internal nostril, nasal respiration evolved for the first time in two lineages of lobe-finned fishes, including the lineage ancestral to tetrapods [103,104]. The presence of ORs in gene families associated with terrestrial species might be explained by tetrapodomorphs adapting to the intertidal olfactory landscape (§3a). The evolution of nasal respiration led to the specialization of the MOS for airborne odours and hence the need for a second, complementary olfactory system for complex odours (§4). For this reason the VNS only appeared in the two groups with nasal respiration—the lungfish and tetrapods (§4), even though vomeronasal receptors had evolved earlier and were found in other lineages [132,134]. Once on land, the hippocampus of terrestrial mammals continued to scaffold memory on olfactory gradients, mediated as the bearing map by the dentate gyrus (§2a,b). Nasal respiration would thus have been fundamental to hippocampal function in mammals (§4). The use of nasal respiration for tracking prey in space may also explain the pattern of hippocampal theta oscillations (§5b). As mammals radiated out of a terrestrial, nocturnal niche into new, extreme environments, where dynamic sniffing and olfactory mapping were no longer possible, directional olfaction was replaced by echolocation, explaining the degeneration of the hippocampus in cetaceans (§5d). Finally, the human external nose may have evolved in the genus, *Homo*, as an adaptation for stereo olfaction, underlining the importance of the hippocampal limbic system and linking the evolution of nasal respiration to hippocampal function in humans (§2c). We should think of the MOS and the hippocampus as a limbic navigational system, a cognitive rhinencephalon (§2b). It encodes a bearing map created from distributed cues, where individual episodic memories (sketch maps) are integrated into the scaffold of the bearing map, recoding and reconciling maps created from discrete and distributed stimuli (§2a). Evolution does not start from scratch—the answer could be obvious as the nose on our face.

In 1962, in an article entitled, ‘The future of data analysis', statistician John Tukey wrote: ‘Far better an approximate answer to the right question, which is often vague, than an exact answer to the wrong question, which can always be made more precise’ [197]. Fundamental problems, such as the nature of memory or the function of the hippocampus, cannot be solved without identifying the right question. The hippocampus is the right question—it plays a key role in spatial memory in vertebrates [198,199]. Spatial orientation is also the right question: it is a first principle that behaviour evolves in response to the distribution of resources in space and time [200], which is why behavioural ecology, having identified its first principles long ago, is so successful [201]. Finally, olfaction is the right question, as the most ancient and universal sensory modality [202] that even today is driving our memory processes. Understanding the evolutionary history of spatial orientation to odours could well provide a blueprint for the evolution of cognition itself [39]. The immunologist Lewis Thomas expressed it this way in 1983: ‘The act of smelling something, anything, is remarkably like the act of thinking itself … ‘ [203, p. 42]. ‘I should think we might fairly gauge the future of biological science, centuries ahead, by estimating the time it will take to reach a complete, comprehensive understanding of odor. It may not seem a profound enough problem to dominate all the life sciences, but it contains, piece by piece, all the mysteries' [203, p. 41].

## Endnote

1 Speculative insights identified by section number.

## Data accessibility

This article has no additional data.
